# Susceptibility to and severity of SARS-CoV-2 infection according to prescription drug use–an observational study of 46,506 Danish healthcare workers

**DOI:** 10.1371/journal.pone.0311260

**Published:** 2024-11-27

**Authors:** Aleksander Eiken, Sofie Bliddal, Marie Villumsen, Kasper K. Iversen, Henning Bundgaard, Rasmus B. Hasselbach, Jonas H. Kristensen, Pernille B. Nielsen, Mia M. Pries-Heje, Andreas D. Knudsen, Kamille Fogh, Jakob B. Norsk, Ove Andersen, Thea K. Fischer, Ram B. Dessau, Sisse R. Ostrowski, Christian Torp-Pedersen, Sisse B. Ditlev, Mikkel Gybel-Brask, Erik Sørensen, Lene H. Harritshøj, Fredrik Folke, Thomas Benfield, Frederik N. Engsig, Henrik E. Poulsen, Henrik Ullum, Ulla Feldt-Rasmussen, Susanne D. Nielsen, Jørgen Rungby

**Affiliations:** 1 Department of Endocrinology, Copenhagen University Hospital (Bispebjerg & Steno Diabetes Center Copenhagen), Copenhagen, Denmark; 2 Department of Medical Endocrinology and Metabolism, Copenhagen University Hospital (Rigshospitalet), Copenhagen, Denmark; 3 Center for Clinical Research and Prevention, Copenhagen University Hospital (Bispebjerg and Frederiksberg Hospital), Copenhagen, Denmark; 4 Department of Cardiology, Copenhagen University Hospital-Herlev and Gentofte, Herlev, Denmark; 5 Department of Clinical Medicine, University of Copenhagen, Copenhagen, Denmark; 6 Department of Emergency Medicine, Copenhagen University Hospital-Herlev and Gentofte, Herlev, Denmark; 7 Department of Cardiology, Copenhagen University Hospital (Rigshospitalet), Copenhagen, Denmark; 8 Faculty of Health and Medical Sciences, University of Copenhagen (KU), Copenhagen, Denmark; 9 Department of Infectious Diseases, Copenhagen University Hospital (Rigshospitalet), Copenhagen, Denmark; 10 Department of Clinical Medicine, Faculty of Health and Clinical Sciences, Copenhagen University, Copenhagen, Denmark; 11 Department of Emergency and Department of Clinical Research, Copenhagen University Hospital–Amager and Hvidovre, Hvidovre, Denmark; 12 Department of Clinical Research, University Hospital of Northern Zealand, Hilleroed, Denmark; 13 Department of Public Health, University of Copenhagen, Copenhagen, Denmark; 14 Department of Clinical Microbiology, Zealand University Hospital, Slagelse, Denmark; 15 Department of Regional Health Research, University of Southern Denmark, Odense, Denmark; 16 Department of Clinical Immunology, Copenhagen University Hospital (Rigshospitalet), Copenhagen, Denmark; 17 Department of Cardiology, Nordsjaellends Hospital, Hillerød, Denmark; 18 Respiratory Research Unit, Department of Respiratory Medicine, Copenhagen University Hospital at Bispebjerg, Copenhagen, Denmark; 19 Copenhagen Center for Translational Research, Copenhagen University Hospital, Bispebjerg and Frederiksberg, Copenhagen, Denmark; 20 Department of Infectious Diseases, Copenhagen University Hospital (Hvidovre), Copenhagen, Denmark; 21 Management Section, Statens Serum Institut (SSI), Copenhagen, Denmark; University of Copenhagen: Kobenhavns Universitet, DENMARK

## Abstract

It is not well investigated whether exposure to specific drug classes is associated with COVID-19. We investigated the risk of SARS-CoV-2 infection and severe COVID-19 among healthcare workers according to prescription drug use. We conducted an observational study among Danish healthcare workers. SARS-CoV-2 positivity was defined as a positive PCR/ELISA test throughout 2020 and severe COVID-19 as any above 48-hour hospitalization within 14 days after infection. Patient characteristics came from online surveys while data on SARS-CoV-2, drugs and hospitalizations came from Danish Health Registers. Infected individuals were matched with uninfected controls based on age, sex, and chronic diseases. Drug exposure was defined as any prescription redemption in the past six and one month(s) before infection for each drug class. Models assessing the risk of infection (conditional logistic regression) and severe COVID-19 (logistic regressions) versus drug usage were adjusted for BMI, smoking, alcohol, education, region, and patient contact when possible. We matched 5,710 SARS-CoV-2-infected cases with 57,021 controls. The odds of infection were reduced by calcium channel blocker (adjusted odds ratio (aOR) 0.81, 95% Confidence Interval (CI): 0.66–1.00) and vasoprotective drug (aOR 0.77, CI: 0.62–0.95) usage during the six months before infection compared to no usage. Exposure to antibacterials in the past month increased the odds of infection (aOR 1.27, CI: 1.09–1.48). Among infected participants, the odds of severe COVID-19 were higher with usage of almost any investigated drug, especially, diuretics (crude odds radio (OR) 4.82, CI:2.15–10.83), obstructive airway disease drugs (OR 4.49, CI: 2.49–8.08), and antibacterials (OR 2.74 CI:1.62–4.61). In conclusion, antibacterials were associated with more SARS-CoV-2 infections and calcium channel blockers with less. Once infected, users of prescription drugs had higher odds of developing severe COVID-19. These findings suggest a need for studies to clarify interactions between specific drug groups, behaviour, known risk factors, and disease susceptibility/severity.

## Introduction

More than six million people have died with severe acute respiratory syndrome coronavirus 2 (SARS-CoV-2) infection during the COVID-19 pandemic [1]. Despite the rapid vaccine development and distribution, the pandemic remains a worldwide major healthcare concern. Therefore, identifying risk factors for SARS-CoV-2 infection and severe COVID-19 disease is essential for improving containment and treatment strategies for at-risk individuals.

Studies of susceptibility to SARS-CoV-2 infection have mainly identified risks related to viral exposure, i.e. traveling in high-risk areas at the beginning of the pandemic, household transmission, social gatherings, and healthcare workers [2–5]. Among healthcare workers, especially those serving in frontline COVID-19 wards were at increased risk of exposure to and infection with SARS-CoV-2 [5, 6]. This makes healthcare workers a group of interest–both to study interindividual differences in susceptibility, and from a societal perspective to improve preventive measures in this important part of the workforce.

The risk of developing severe COVID-19 (hospitalization or death) has been associated with multiple risk factors including male sex, age, BMI, and comorbidities such as chronic obstructive pulmonary disease, cardiovascular disease, diabetes, and hypertension [7–10]. However, drug exposure could affect susceptibility to SARS-CoV-2 infection and severity of COVID-19 via several plausible mechanisms; impact of angiotensin-converting enzyme inhibitors on the angiotensin-converting enzyme 2 as the entry receptor for SARS-CoV-2 [11, 12], prevention from calcium channel blockers of viral cell entry via calcium ions’ modulation of host-pathogen interaction [13], and corticosteroids as potential inhibitors of viral replication and cytokine production associated with hyperinflammation and mortality [14–16]. Nevertheless, studies of prescription drugs such as renin-angiotensin inhibitors or corticosteroids have shown mixed results with regards to disease severity and outcome [17–20], and most studies of prescription drug use have focused on the risk of severe disease and death rather than the risk of contracting SARS-CoV-2 [21–23]. Hence, the aim of this study was to investigate whether prescription drug use was associated with SARS-CoV-2 infection among healthcare workers and, among those infected, whether such use was associated with severe COVID-19.

## Materials and methods

### Study design and participants

In an observational study, all healthcare workers from the Capital Region and Region Zealand in Denmark were invited to participate in a study on SARS-CoV-2 among healthcare workers with three rounds of questionnaires and blood sampling a few months apart (between April 2020 and December 2020). As previously described [5], participants filled in an online questionnaire and a blood sample was drawn with the purpose of enzyme-linked immunosorbent assay (ELISA) SARS-CoV-2 testing in each round. Inclusion criteria for the present study were: Participant consent, participation in at least one of the questionnaire rounds, and at least one conclusive SARS-CoV-2 test (either Polymerase Chain Reaction (PCR) or ELISA) prior to January 1^st^ 2021.

### Questionnaires

All participants filled in a detailed online questionnaire on background data (se variables under section ‘statistics’) and self-reported chronic diseases including asthma, other lung diseases than asthma, heart disease, hypertension, kidney disease, diabetes, immunodeficiencies or ‘other chronic disease’. Only answers from the first questionnaire from each participant was used in this study. To avoid missing values, participants were unable to skip most questions (i.e. self-reported disease status) and whenever possible questionnaire answers were validated programmatically before submission. Obvious typos in the answers were corrected manually before analysis, however when in doubt the value of concern was replaced by ‘missing’.

### SARS-CoV-2 positivity and COVID-19 severity

At each questionnaire round, blood samples were obtained for ELISA SARS-CoV-2 antibody testing. As previously described in detail [24], the ELISA kit from Wantai (Beijing, China) was applied manually on all samples according to the manufacturer’s instructions. Internal validations showed a sensitivity of 96.7% and a specificity of 99.5%. Furthermore, information on PCR tests for SARS-CoV-2 prior to January 1^st^, 2021 was obtained from the national surveillance by the Danish Health Authorities (Statens Serum Institut) with data from the Danish Microbiology Database [25]. Hence, dates of PCR tests were scattered throughout 2020 and not tied to a questionnaire round but performed for various reasons including COVID-19 symptoms and later in 2020 also prior to specific events or as regular screenings for certain employees. The participants were considered SARS-CoV-2 positive if they had a positive ELISA and/or PCR test at any time prior to January 1^st^, 2021. The date of infection was defined as the date of the first positive SARS-CoV-2 test and succeeding positive tests were not considered. Based on information from the National Patient Register, severe COVID-19 disease was defined as any hospital admission lasting over 48 hours with time of admission within 14 days from the date of infection. Furthermore, to ensure the infection was present, SARS-CoV-2 infection had to be confirmed via a PCR.

### Matching

For the analysis the assessing risk of infection according to medication exposure, we used a case-control design: Prior to analysis, all SARS-CoV-2 positive cases were matched with ten controls sampled among participants with no or only negative SARS-CoV-2 tests as of the date of infection for the case. As potential matches were sampled from all participants, who were COVID-19 negative (or had had no test) at the date of infection for the case, participants (also those with a later positive test) could be included as controls multiple times. Exact matching was used, and matching variables were sex (male = 1, female = 0), age (<30, 30–50 or >50 years), and specific self-reported chronic diseases (asthma, diabetes, lung disease other than asthma, heart disease, kidney disease, hypertension or weakened immune system). All disease groups were binary ’yes-no’. SARS-CoV-2 infected cases and matched controls were excluded from analysis if four or less controls were available.

For the analysis assessing the risk of severe COVID-19 according to medication exposure, we compared severe COVID-19 cases to mild COVID-19 cases and hence no matching was performed. However, to avoid selection bias, mild cases of SARS-CoV-2 based on ELISA testing were excluded, as severe COVID-19 required a positive PCR test.

### Register data

In the Danish Prescription Register, every redemption in Denmark is registered based on the Anatomical Therapeutic Chemical Classification System (ATC codes) as defined by the World Health Organization [26]. Table 1 provides an overview of the drug classes included in this study. Drug selection was based on data availability within the register and a consensus decision within our research group to select drug classes frequently used to either treat the self-reported chronic diseases of interest or to have potential modulatory effects on the immune system.

**Table 1:** ATC codes and subgroups for included prescription drugs.

ATC level 1	Name	ATC level 2	Name
A	Alimentary tract and metabolism	A07	Antidiarrheals, intestinal antiinflammatory/antiinfective agents
C	Cardiovascular system	C01	Cardiac therapy
		C03	Diuretics
		C05	Vasoprotectives
		C07	Beta blocking agents
		C08	Calcium channel blockers
		C09	Agents acting on the renin-angiotensin system
		C10	Lipid modifying agents
H	Systemic hormonal preparations	H02	Corticosteroids for systemic use
J	Anti-infectives for systemic use	J01	Antibacterials for systemic use
		J02	Antimycotics for systemic use
		J05	Antivirals for systemic use
		J07	Vaccines
R	Respiratory system	R03	Drugs for obstructive airway diseases
N	Nervous system	N05	Psycholeptics
		N06	Psychoanaleptics

Overview of the included prescription drug type groups according to the Anatomical Therapeutic Chemical Classification System (ATC codes) as defined by the World Health Organization [26].

Based on the Danish Civil Registration system, in which every Dane has a unique identification number (Central Person Register (CPR) number), self-reported questionnaire information was linked to Danish Medical Register data. For both SARS-CoV-2 positive cases and matched controls, information on redeemed prescriptions (date of pick-up from a pharmacy) was obtained from the Danish Register of Medicinal Product Statistics from 187 days to seven days (six months) or 37 days to seven days (one month) prior to the date of infection for the case. A full seven-day lag period was included to ensure medication exposure came prior to the SARS-CoV-2 infection. Within these case/control specific time frames, prescription drug use (’yes-no’ for each ATC code) was defined as the occurrence of any redeemed prescription within the ATC codes of interest. The time frame of six months was chosen to most precisely include subjects with current medication usage within long-term usage drugs for chronic diseases while excluding patients with no or only previous exposure. The time frame of one month was chosen to most precisely include subjects with recent medication usage within drugs which are only administered for a short period of time (i.e. antibacterials).

Hospital admission data was obtained from the National Patient Register 3 (LPR3) to identify participants with severe COVID-19. For the analyses related to severe COVID-19, prescription drug use was also defined as described above. All statistical analyses were conducted based on redemptions of prescription drugs assessed at ATC level 2 (e.g. therapeutic subgroup).

### Statistics

Self-reported questionnaire data was linked to the results from SARS-CoV-2 tests, hospital admissions, and prescription drug pick-up date using CPR numbers.

Results were given as median and interquartile range (IQR) or absolute numbers (n) and percentages (%). Categories with less than five participants, however not zero, were reported as ‘1–4’ to protect anonymity, as required by Statistics Denmark. For comparison of demographic data, Chi Squared and Fisher’s exact tests were used depending on sample size.

In the analysis of the odds of SARS-CoV-2 infection according to prescription drug use, a single conditional logistic regression model with all included prescription drug categories for the prior six months was used. The model was checked for multicollinearity through calculation of the variance inflation factor with removal of variables with values above five. The analysis was adjusted for self-reported, questionnaire obtained information on body mass index (BMI) (<18.5, 18,5–25,25–30, >30 kg/m^2^, missing), smoking status (yes, no, former smoker, missing), alcohol intake (0, 0–7, 7–15, >15 units per week, missing), educational level (none or short, middle, long, missing), patient contact (none, partly, full time, missing), and place of living (Capital Region or Region Zealand).

The primary analysis was conducted using six months of prescription data and all SARS-CoV-2 positive cases based on both ELISA and PCR testing. Sensitivity analyses were performed for prescriptions redeemed within only one month prior to infection, due to short treatment duration of certain drug types. As ELISA testing may remain positive also after active infection has ceased, additional sensitivity analyses with one- and six-months medication exposure for PCR positive cases only were also conducted. A sensitivity analysis was also performed with exclusion of SARS-CoV-2 positive patients, who tested positive prior to filling out the questionnaire due potential recall-bias issues.

In analyses of the odds of severe COVID-19 according to prescription drug use or no use, an unadjusted logistic regression model was conducted for each prescription drug independently. This was necessary because the number of observations was too small to allow analysis in the same model. In each model, medication usage for cases with severe COVID-19 was compared to medication usage for those with mild, PCR verified COVID-19. Furthermore, analyses were only performed if there were five or more participants who had been exposed to the prescription drug in question and had had severe COVID-19. For prescription drugs where the number of exposed participants with severe COVID-19 was above 15, adjusted sensitivity analyses were performed separately for each of the following covariates, as numbers did not allow adjustment for all in the same model: any chronic disease (yes/no), age, sex and BMI [27]. We also conducted a sensitivity analysis assessing the odds of severe COVID-19 for users of any included drug (drug use/no drug use), which allowed adjustment for age, BMI and chronic disease (yes/no).

Odds ratios (OR) and adjusted odds ratios (aOR) were reported with 95% Confidence intervals (CI), and a result was considered significant with p < 0.05. All statistics were carried out in R version 4.0.3 via the base functions and packages ‘finalfit v1.0.2’, ‘car v 3.0.10’, ‘survival v 3.6–4’ and ‘epiDisplay 3.5.0.1’ [28].

### Ethics

This study based on questionnaire and register data was evaluated by the Regional Scientific Ethics Committee of the Capital Region, and the Committee concluded that the study did not require a scientific ethics approval (Jnr-H-20026288). However, all participants were asked in the questionnaire to consent or decline the extraction of registry data, and these answers were respected (46 declined). The study was approved by the data responsible institution (the Capital Region of Denmark (Approval number: P-2019-191)) in accordance with the General Data Protection Regulation (GDPR).

## Results

**Fig 1:**
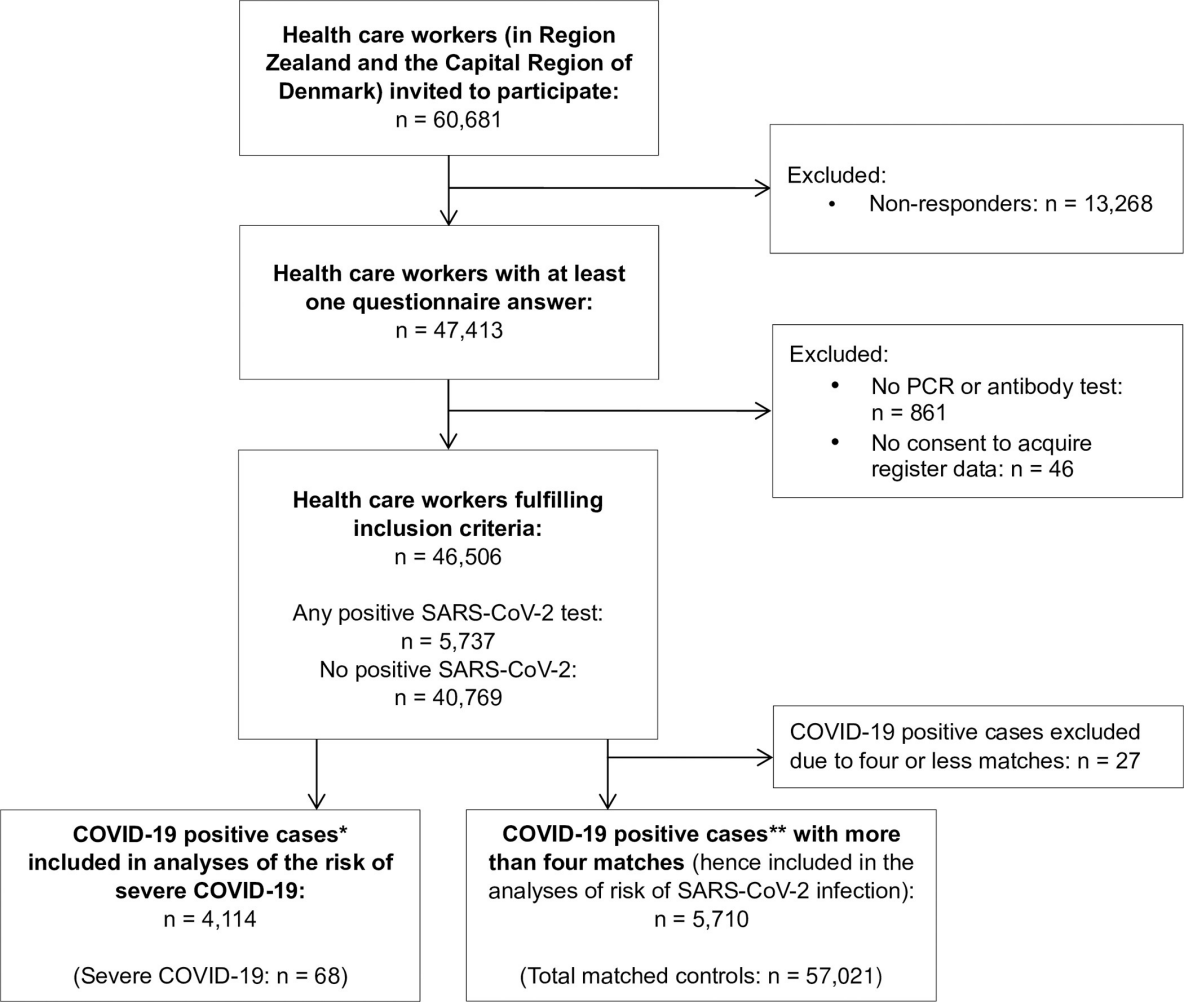
Flowchart of the inclusion process. Flowchart of the inclusion process of healthcare workers. *Defined based on PCR tests only. Severe COVID-19 was defined as any hospital admission longer than 48 hours with time of admission within 14 days after the positive SARS-CoV-2 test result. **Defined based on PCR tests and ELISA tests. Abbreviations: PCR, polymerase chain reaction. SARS-CoV-2, Severe Acute Respiratory Syndrome Coronavirus-2.

A total of 46,506 healthcare workers answered at least one questionnaire between April 7^th^, 2020 and December 31^st^, 2020 and were eligible for inclusion (Fig 1). The first questionnaire from each participant was filled in during the first screening round (April 4^th^ to May 19^th^, 2020) by 81.9% of the participants, the second screening round (June 2^nd^ to June 25^th^, 2020) by 9.2%, and the third round (September 15^th^ to October 27^th^, 2020) by 6.7%. The remaining 2.2% filled in the questionnaires outside of the designated screening rounds, but all prior to January 1^st^, 2021. All participants were tested for SARS-CoV-2 at least once prior to January 1^st^, 2021, as required for inclusion. Most individuals were tested several times and with both ELISA and PCR tests. 45,606 (98.0%) individuals had one or more ELISA test results, and 45,664 (98.2%) individuals had one or more PCR test results.

### SARS-CoV-2 infection

SARS-CoV-2 was detected in 5,737 (12.3%) participants of whom 2,092 had at least one prescription redemption during the six months prior to the infection. SARS-CoV-2 was detected via PCR as the first positive test method for 4,114 (71.7%) individuals and ELISA testing for the remaining. The first positive test was detected on March 8^th^, 2020, while the last positive test was detected on December 31^st^, 2020.

### Matching and characteristics

It was possible to match 5,710 (99.5%) SARS-CoV-2 positive individuals with 5 or more controls while 27 SARS-CoV-2 positive individuals were excluded as matching was not possible. Lack of matches were mainly due to infrequent combinations of self-reported diseases. Ten controls were found for 5,685 (99.1%) and the total number of controls was 57,021. Amongst the sampled controls, 15,537 participants occurred only one time while 9,529 occurred two, 4,186 occurred three, 1,549 occurred four, 488 occurred five, 135 occurred six, 42 occurred seven and 16 occurred eight times, respectively. 3,939 cases figured once or more as controls prior to their positive COVID-19 test.

The cases and controls were successfully matched on sex and age distribution (Table 2). The cases had a statistically higher BMI than controls and a slightly higher proportion of cases were non-smokers. No difference was found within consumption of alcohol with most of both groups reporting 7 or fewer units per week. However, more cases had a lower educational level compared to controls, and a higher proportion of cases reported having full patient contact in their work. Among the included participants, 2,058 (36.0%) of the cases and 20,723 (36.3%) of the controls had at least one prescription redemption in the six months timeframe of interest. The proportion of SARS-CoV-2 positive cases was higher for those working in the Capital Region of Denmark. The most prominent self-reported diseases, distributed equally within both cases and controls due to matching, were asthma (7.3%) and hypertension (6.2%), while the remaining chronic diseases were rare (<2%) especially kidney disease and ‘other lung disease than asthma’, both with a prevalence of <0.5%. Reporting of ‘other chronic disease’ varied slightly amongst cases (6,5%) and controls (6.9%).

**Table 2:** Characteristics of cases and controls according to SARS-CoV-2 infection.

Variable		Total	SARS-CoV-2*	
			Negative (n, %)	Positive (n, %)
**N**	**-**	62,731	57,021 (100.0%)	5,710 (100.0%)
**Sex (matching variable)**	**Female**	49,316	44,829 (78.6%)	4,487 (78.6%)
	**Male**	13,415	12,192 (21.4%)	1,223 (21.4%)
**Age, years (matching variable)**	**<30**	15,509	14,097 (24.7%)	1,412 (24.7%)
	**30–50**	28,029	25,478 (44.7%)	2,551 (44.7%)
	**>50**	19,193	17,446 (30.6%)	1,747 (30.6%)
**BMI (kg/m** ^**2**^ )**	**< 18,5**	1,164	1,073 (1.9%)	91 (1.6%)
	**18,5–25**	35,232	32,132 (56.4%)	3,100 (54.3%)
	**25–30**	17,052	15,442 (27.1%)	1,610 (28.2%)
	**> 30**	7,991	7,189 (12.6%)	802 (14.0%)
	**Missing**	1,292	1,185 (2.1%)	107 (1.9%)
**Smoking**	**Non-smoker**	48,499	43,927 (77.0%)	4,572 (80.1%)
	**Former smoker**	3,525	3,208 (5.6%)	317 (5.6%)
	**Smoker**	9,435	8,758 (15.4%)	677 (11.9%)
	**Missing**	1,272	1,128 (2.0%)	144 (2.5%)
**Alcohol intake, units per week**	**0**	21,113	19,173 (33.6%)	1,940 (34.0%)
	**1–7**	31,962	29,137 (51.1%)	2,825 (49.5%)
	**8–14**	5,715	5,167 (9.1%)	548 (9.6%)
	**≥15**	771	702 (1.2%)	69 (1.2%)
	**Missing**	3,170	2,842 (5.0%)	328 (5.7%)
**Educational level**	**Short or none**	6,406	5,679 (10.0%)	727 (12.7%)
	**Middle**	36,806	33,465 (58.7%)	3,341 (58.5%)
	**Long**	16,544	15,224 (26.7%)	1,320 (23.1%)
	**Missing**	2,975	2,653 (4.7%)	322 (5.6%)
**Patient contact**	**No**	9,187	8,579 (15.0%)	608 (10.6%)
	**Yes–partly**	11,812	10,773 (18.9%)	1,039 (18.2%)
	**Yes**	41,589	37,537 (65.8%)	4,052 (71.0%)
	**Missing**	143	132 (0.2%)	11 (0.2%)
**Region**	**Capital**	49,611	45,000 (78.9%)	4,611 (80.8%)
	**Zealand**	13,120	12,021 (21.1%)	1,099 (19.2%)
	**Missing**	0	0	0

*Both positive ELISA test and PCR test results were included.

**According to BMI categories as defined by the World Health Organization.

Abbreviations: BMI, body mass index. SARS-CoV-2, Severe Acute Respiratory Syndrome Coronavirus-2.

### Drug use and odds of SARS-CoV-2 infection

**Fig 2:**
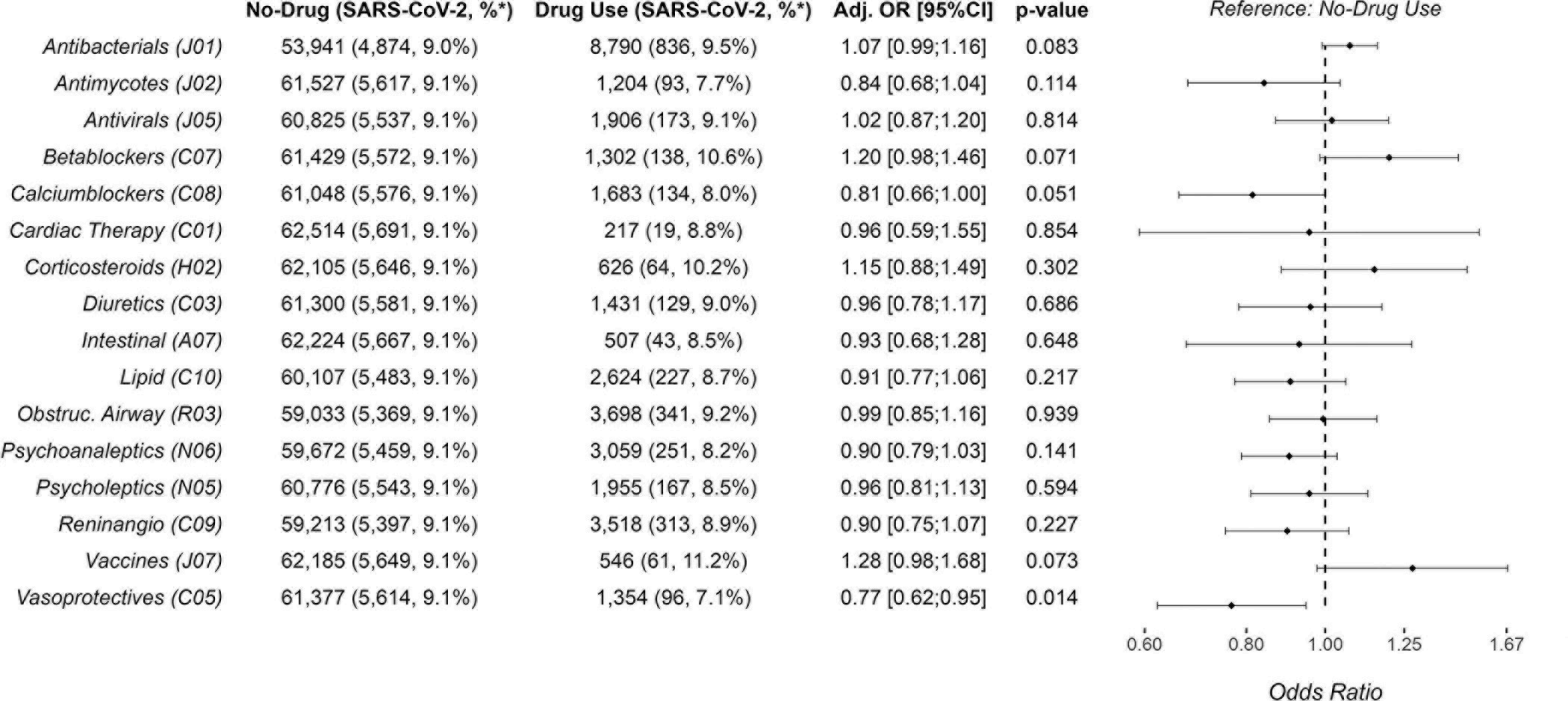
Adjusted odds ratios of SARS-CoV-2 positivity according to prescription drug use. Adjusted odds ratios for having a positive SARS-CoV-2 test (PCR or ELISA) according to use of prescription drugs (yes/no) in the six months before date of infection, compared to controls matched on sex, age and chronic diseases. Prescription drug categories are based on the Anatomical Therapeutic Chemical Classification System as defined by the World Health Organization (codes are listed in parenthesis). The analysis was adjusted for body mass index (BMI) (<18.5, 18,5–25,25–30, >30 kg/m^2^, missing), smoking (yes, no, former smoker, missing), alcohol intake (0, 0–7, 7–15, >15 units per week, missing), educational level (none or short, middle, long, missing), patient contact (none, partly, full time, missing), and region of employment (Capital Region or Region Zealand). *) Indicates SARS-CoV-2 positive fraction in percent of drug users and no-drug users, respectively. Abbreviations: Adj., adjusted. OR, odds ratio. PCR, polymerase chain reaction. SARS-CoV-2, Severe Acute Respiratory Syndrome Coronavirus-2.

Participants receiving calcium channel blockers or vasoprotective drugs in the previous 6 months had lower odds (aOR: 0.81, 95% CI: 0.66–1.00; 0.77, 95% CI: 0.62–0.95, respectively) of having a positive test for SARS-CoV-2 compared to those not taking these drugs. (Fig 2). Antibacterials were the most common prescription drug class and use in the previous 6 months were positively associated with a positive SARS-COV-2 test (aOR: 1.07, 95% CI: 0.99–1.16). Similarly, use of betablockers and vaccines in the previous 6 months was positively associated with a positive SARS-CoV-2 test. The remaining prescription drugs were not associated with SARS-CoV-2 infection.

In the sensitivity analysis including only PCR positive tests (S1 Fig) and six months of prescription data, use of calcium channel blockers remained associated with lower odds of a positive SARS-CoV-2 test result (aOR: 0,75 95% CI: 0.59–0.96) while the protective effect of vasoprotective drugs was less prominent. Use of antibacterials was still associated with increased odds of a positive SARS-CoV-2 test result (aOR: 1.07 95% CI: 0.98–1.18), however the effect of betablockers was less prominent. Interestingly, when considering only prescription drugs used during the one month prior to the infection, use of antibacterials was more pronouncedly associated with increased odds of a positive SARS-CoV-2 test result. This was the case both when considering all positive test results (S2 Fig) or PCR test results only (S3 Fig). No other drug in the sensitivity analyses for the past one month (including calcium channel blockers, vasoprotective- and antimycotic drugs) was associated with SARS-CoV-19 infections. In general, results were similar when considering only individuals filling out the questionnaire before being tested positive (S4 Fig).

### Severe COVID-19

Among the 4,114 cases who tested positive for SARS-CoV-2 via PCR tests, 68 (1.7%) had severe COVID-19. Compared to those with mild COVID-19 based on PCR tests only, participants with severe COVID-19 were older (>50 years of age), more often male, more often had a BMI above 30 kg/m^2^ and had a higher alcohol consumption (Table 3). No other demographic characteristic was associated with severe COVID-19. Among those with severe COVID-19, half (52.9%) reported suffering from a chronic disease while only one out of five (21.9%) of those with mild COVID-19 reported having a chronic disease (see Table 3).

**Table 3:** Characteristics of participants according to COVID-19 severity based on PCR testing only.

Variable		COVID-19*		
		Total, n	Mild (n, %)	Severe (n, %)
**N**		4,114	4,046 (100.0%)	68 (100.0%)
**Sex**	**Female**	3,279	3,236 (80.0%)	43 (63.2%)
	**Male**	835	810 (20.0%)	25 (36.8%)
**Age**	**<30 years**	865	860 (21.3%)	5 (7.4%)
	**30–50 years**	1,947	1,928 (47.7%)	19 (27.9%)
	**>50 years**	1,302	1,258 (31.1%)	44 (64.7%)
**BMI (kg/m** ^**2**^ )**	< 25 or missing***	2,286	2,260 (55.9%)	26 (38.2%)
	**25–30**	1,193	1,168 (28.9%)	25 (36.8%)
	**> 30**	635	618 (15.3%)	17 (25.0%)
**Smoking**	**Missing**	107	107 (2.6%)	0
	**Non-smoker**	3,267	3,209 (79.3%)	58 (85.3%)
	**Smoker or former smoker**	740	730 (18.0%)	10 (14.7%)
**Alcohol intake**	0 or missing***	1,737	1,714 (42.4%)	23 (33.8%)
	**1–7**	1,966	1,939 (47.9%)	27 (39.7%)
	**≥8**	411	393 (9.7%)	18 (26.5%)
**Educational level**	None, short or missing***	674	664 (16.4%)	10 (14.7%)
	**Middle**	2,473	2,434 (60.2%)	39 (57.4%)
	**Long**	967	948 (23.4%)	19 (27.9%)
**Patient contact**	**Missing**	6	6 (0.1%)	0
	**No**	415	407 (10.1%)	8 (11.8%)
	**Yes–partly**	711	694 (17.2%)	17 (25.0%)
	**Yes**	2,982	2,939 (72.6%)	43 (63.2%)
**Region**	**Missing**	0	0	0
	**Capital**	3,320	3,261 (80.6%)	59 (86.8%)
	**Zealand**	794	785 (19.4%)	9 (13.2%)
**Asthma**	**No**	3800	3,746 (92.6%)	54 (79.4%)
	**Yes**	314	300 (7.4%)	14 (20.6%)
**Hypertension**	**No**	3,840	3,785 (93.5%)	55 (80.9%)
	**Yes**	274	261 (6.5%)	13 (19.1%)
Other Chronic Disease****	**No**	3,842	3,782 (93.5%)	60 (88.2%)
	**Yes**	272	264 (6.5%)	8 (11.8%)
**Any Chronic Disease**	**No**	3,193	3,161 (78.1%)	32 (47.1%)
	**Yes**	921	885 (21.9%)	36 (52.9%)

* COVID-19 cases based on PCR testing only. Severe COVID-19 was defined as any hospital admission for more than 48 hours with time of admission within 14 days after a positive SARS-CoV-2 PCR test.

**According to BMI categories as defined by the World Health Organization.

*** As there were between 1–4 missing, groups were merged to allow display of data according to Statistics Denmark’s guidelines.

****’Other Chronic Disease’ includes ’Weakened Immune System’, ’Kidney Disease’, ’Heart Disease’, ’Lunge Disease Other Than Asthma’ and ’Diabetes’

Abbreviations: BMI, body mass index. SARS-CoV-2, Severe Acute Respiratory Syndrome Coronavirus-2. PCR, polymerase chain reaction.

**Fig 3:**
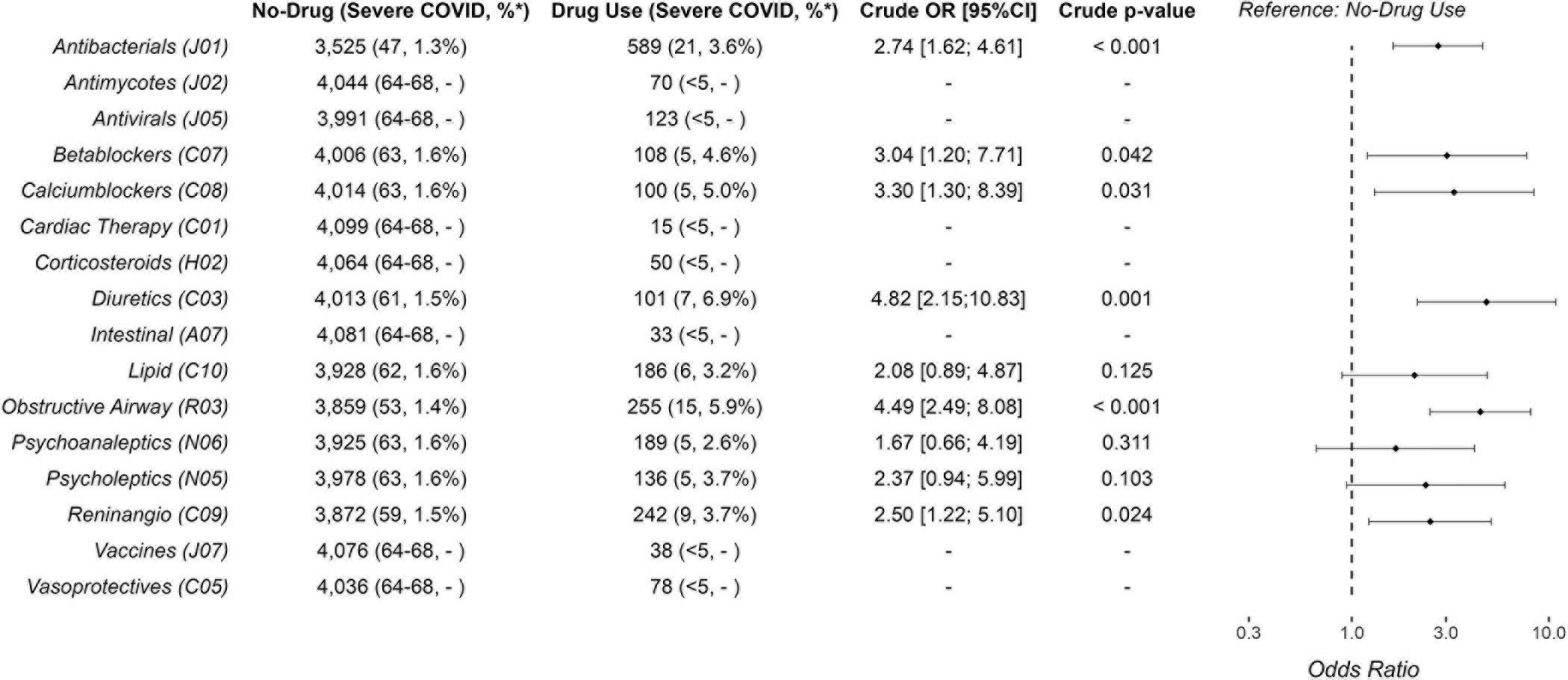
Odds ratios of severe COVID-19 according to prescription drug use among 4,114 infected participants. Crude odds ratios of a severe COVID-19 infection when comparing use versus no use (reference) of prescription drugs in the six months prior to a PCR SARS-CoV-2 positive test. Severe COVID-19 was defined as hospitalization for more than 48 hours with time of admission within 14 days of a positive SARS-CoV-2 PCR test.

In the subgroups with enough participants to conduct analyses, those with use of antibacterials, diuretics, betablockers, calcium channel blockers, drugs for obstructive airway disease and renin–angiotensin medication had increased odds of severe COVID-19 (Fig 3) compared to those with no use for the past six months. There were enough users of antibacterials and drugs for obstructive airway disease to allow analyses with adjustment for age, sex, any chronic diseases (yes/no), or BMI (see S1-S9 Tables in S1 File). Only adjustment for any chronic diseases changed the results and, thus, the odds of severe COVID-19 in users of drugs for obstructive airway disease decreased but remained significantly higher (crude OR: 4.49, 95% CI: 2.49–8.08, and aOR: 2.31, 95% CI: 1.21–4.42, see S2 Table in S1 File). When assessing the odds of severe versus mild COVID-19 in the sensitivity analysis considering users of any drugs, those with drug use had higher odds of severe COVID-19 (crude OR 3.48 95% CI: 2.10–5.78) also after adjusting for age, BMI and any chronic diseases (aOR: 2.04, 95% CI: 1.16–3.59, see S9 Table in S1 File).

## Discussion

In Danish healthcare workers, the odds of being infected with SARS-CoV-2 were lower in those with usage of calcium channel blockers and vasoprotective drugs in the past six months prior to infection compared to matched controls, and higher for those who had used antibacterials, especially within the month before infection. Other investigated prescription drugs were not associated with SARS-CoV-2 infection. Once infected, the odds of severe COVID-19 were higher for COVID-19 cases with use of antibacterials, beta-blockers, calcium channel blockers, diuretics, angiotensin receptor blockers/ACE inhibitors, and drugs for obstructive airway disease in the past six months, compared to COVID-19 cases with no use of such drugs.

Users of antibacterials in the past month had increased odds of SARS-CoV-2 infection with results being consistent based on analysis with ELISA and PCR or PCR tests only. The same pattern, however, slightly less prominent, was observed when assessing antibacterial usage for the past six months. Previous studies have demonstrated high antibacterial prescription rates for (viral) upper respiratory tract infections [29], and higher antibacterial prescription rates during the pandemic [30, 31], however a more liberal use of antibacterials cannot explain the association with subsequent SARS-CoV-2 infections. We speculate that this finding could reflect a more general immunosuppression among individuals taking antibacterials in the months preceding SARS-CoV-2 infection, possibly through modulation of microbiota, which seems to be important for regulating immune response to viral lung infections [32–35]. However other reasons could explain the association including simply increased exposure to infectious agents in this group.

Contrary to use of antibacterials, users of calcium channel blockers consistently had reduced odds of SARS-CoV-2-infections, whereas other anti-hypertensive drugs were not associated with infections. In a recent meta-analysis of hypertensive drugs [36] there was an unaltered incidence of SARS-CoV-2 among users of any antihypertensive drugs, which supports the findings of the present study except for that of calcium channel blockers. Other studies have shown an anti-viral effect of calcium channel inhibitors on a cellular level resulting in reduced viral entry, replication, and thus progeny [13, 37]. Thus, whether calcium channel blockers do indeed reduce SARS-CoV-2 incidences remains a question for further research.

Most of the studied drugs, including inhaled corticosteroids, were not associated with infections. Previous studies have associated inhaled corticosteroids with a higher prevalence of pneumonia in both chronic obstructive pulmonary disease and to a lower extent asthma [38, 39]. However, a previous study found a lower prevalence of SARS-CoV-2-positivity among individuals with chronic respiratory disease compared to the general population [40]. Thus, our finding of no association between inhaled corticosteroids and SARS-CoV-2 infection even after matching for disease status could potentially reflect the balance between an a priori increased susceptibility in patients with inhaled corticosteroids, as previously found [38, 39], and those at-risk patients with severe disease practicing extensive social distancing during the first period of the COVID-19 pandemic [41, 42]. Indeed, even though matching was based on sex, age and disease status, we were not able to match on disease severity nor social distancing, thus medication usage could to some extent be proxies for these potential confounders. This is in line with reports of various patient groups with chronic diseases experiencing a high level of fear, anxiety and/or depression during the beginning of the pandemic [43, 44], which may be even more pronounced among healthcare workers who had a high prevalence of mental health concerns during the pandemic [45, 46]. Thus, social distancing could modulate the odds of infection for healthcare workers with need of prescription drugs rather than an actual mechanism related to the drug itself.

Once infected, users of prescription drugs had higher odds of severe COVID-19 which was a general finding rather than related to subtypes of drugs. Contrary to this, a large international cohort analysis found no increased risk of hospitalization or death among 1,355,349 unselected users of antihypertensive drugs when considering patient characteristics including age, sex, other demographics, and previous conditions [47]. In a study by Fosbøl et al. [48] users of angiotensin-converting enzyme inhibitors or angiotensin receptor blockers more often died or developed severe COVID-19 compared to non-users (31.9% vs 14.2%), however, this difference was not significant when adjusting for age, sex and medical history (adjusted hazard ratio 1.04, 95% CI: 0.89–1.23). The same lack of association with infection and severe disease has been demonstrated in a range of studies investigating a multitude of prescription drug categories [49]. Thus, our finding of increased odds of severe COVID-19 among prescription drug users is most likely explained by the users’ a priori worse constitution rather than a risk associated with the drugs themselves. However, in the analyses adjusted for presence of any chronic disease the odds remained higher (although less so) among users of prescription drugs and drug use could therefore not only be interpreted as a proxy for chronic disease.

This study provides new insights related to disease susceptibility among healthcare workers. Participants with the lowest educational level and with the highest degree of patient contact, respectively, had a significantly higher prevalence of SARS-CoV-2 infection, but these factors were not associated with an increased prevalence of severe COVID-19. Improved measures to protect healthcare workers against infectious diseases including educational initiatives on SARS-CoV-2 prevention for those with patient contact seem to be an important aspect of healthcare planning in the battle against COVID-19.

### Strengths and limitations

The present study is a large observational study with validated laboratory data on SARS-CoV-2 status in combination with detailed self-reported questionnaire data from all participants and national register data. All prescription drug redemptions in Denmark are registered by law in the Danish Register of Medicinal Product Statistics making data highly reliable [50]. This made it possible to study the use of prescription drug use prior to the SARS-CoV-2 diagnosis. Furthermore, based on the questionnaire data we were able to match almost all cases 1:10 with controls based on sex, age, and disease status and furthermore adjust for multiple confounders known to impose a risk of SARS-CoV-2 (BMI, smoking, alcohol consumption, educational level, patient contact, and place of living). Indeed, based on our data collection, exposures (drug redemptions) were present at the time of our outcome (infection), which further strengthens our results.

Nevertheless, there are likely other confounding factors not addressed as illustrated by the finding that use of vasoprotective drugs was associated lower prevalence of SARS-CoV-2 infections. Vasoprotective drugs were mainly local treatments for haemorrhoids. The association was not replicated in the sensitivity analysis regarding prescription redemption within the past month leading up to infection, which makes it less likely, that these drugs were in fact protective. This is further substantiated by the fact that the active drugs (lidocaine and fluocortolone) have a short half time of a few hours [51, 52], and that the treatment is not prescribed for continuous use and therefore likely to be used shortly after redemption. A long-term protective effect of vasoprotective drugs by e.g. the absorbed corticosteroids also seems unlikely as corticosteroids for systemic use were not protective.

Furthermore, the limited number of participants with severe COVID-19 using prescription drugs hindered adjusted analyses for comorbidity or demographic factors, which in the demographic analyses were significantly associated with higher odds of severe COVID-19 (BMI and alcohol intake). The admitted participants, whom we considered infected with severe COVID-19, may also have had additional acute conditions partly or fully responsible for the admission, and adjustment for this was also not possible. Thus, the results regarding odds of severe COVID-19 should be interpreted with caution. The risk of confounding by indication is reflected by a reduction in the odds estimate of severe COVID-19 for both antibacterials and drugs for obstructive airway disease when adjusting for the presence of chronic disease. On the other hand, this cannot fully explain the associations, as in both cases, the odds of infection remained significantly higher despite the adjustment for chronic disease.

Another limitation of the study was the lack of information on treatment duration and compliance, details of hospital admissions, or prolonged symptoms or sequelae (‘long COVID’ [53]). Furthermore, even though a high proportion of the invited individuals submitted questionnaire answers (47,413 out of 60,681, see Fig 1), we cannot rule out selection bias within our studied population and memory bias within the replies to the questionnaires. Furthermore, we cannot rule out, that a few individuals may have had only an early negative PCR or ELISA test and a subsequent SARS-CoV-2 infection without any later positive test results and hence contributing to an underestimation of SARS-CoV-2 positive individuals. However, as most individuals had multiple tests, this most likely seems a negligible issue.

## Conclusion

Among 5,710 SARS-CoV-2 positive healthcare workers matched with 57,021 negative controls, SARS-CoV-2 infection varied depending on prescription drug use. In particular, those with recent antibacterial use were more likely to have a positive SARS-CoV-2 test, while those with use of calcium channel blockers were less likely to have a positive SARS-CoV-2 test. Once infected, use of almost any of the investigated prescription drugs increased the odds of developing severe COVID-19. The findings suggest a need for more mechanistic studies to clarify interactions between specific drug groups, behaviour, known risk factors and disease susceptibility/severity.

## Supporting information

S1 Fig Sensitivity analysis of the odds ratios of SARS-CoV-2 positivity (PCR only) according to the past six months’ prescription drug use. Adjusted odds ratios of having a positive SARS-CoV-2 test (PCR only) according to use of prescription drugs (yes/no) in the six months before date of infection, compared to negative controls matched on sex, age and chronic disease. Prescription drug categories are based on the Anatomical Therapeutic Chemical Classification System as defined by the World Health Organization (codes are listed in parenthesis). The analysis was adjusted for body mass index (BMI) (<18.5, 18,5–25,25–30, >30 kg/m^2^, missing) smoking (yes, no, former smoker, missing), alcohol intake (0, 0–7, 7–15, >15 units per week, missing), educational level (none or short, middle, long, missing), patient contact (none, partly, full time, missing), and place of living (Capital Region or Region Zealand). *) Indicates SARS-CoV-2 positive fraction in percent of drug users and no-drug users, respectively. Abbreviations: Adj., adjusted. OR, odds ratio. PCR, polymerase chain reaction. SARS-Cov-2, Severe Acute Respiratory Syndrome Coronavirus-2.
(TIF)

S2 Fig Sensitivity analysis of the odds ratios of SARS-CoV-2 positivity (PCR or ELISA) according to the past one month’s prescription drug use. Adjusted odds ratios of having a positive SARS-CoV-2 test (PCR or ELISA) according to use of prescription drugs (yes/no) in the month before date of infection, compared to negative controls matched on sex, age and chronic disease. Prescription drug categories are based on the Anatomical Therapeutic Chemical Classification System as defined by the World Health Organization (codes are listed in parenthesis). The analysis was adjusted for body mass index (BMI) (<18.5, 18,5–25,25–30, >30 kg/m^2^, missing) smoking (yes, no, former smoker, missing), alcohol intake (0, 0–7, 7–15, >15 units per week, missing), educational level (none or short, middle, long, missing), patient contact (none, partly, full time, missing), and place of living (Capital Region or Region Zealand). *) Indicates SARS-CoV-2 positive fraction in percent of drug users and no-drug users, respectively. Abbreviations: Adj., adjusted. OR, odds ratio. PCR, polymerase chain reaction. SARS-Cov-2, Severe Acute Respiratory Syndrome Coronavirus-2.
(TIF)

S3 Fig Sensitivity analysis of the odds ratios of SARS-CoV-2 positivity (PCR only) according to the past month’s prescription drug use. Adjusted odds ratios of having a positive SARS-CoV-2 test (PCR only) according to use of prescription drugs (yes/no) in the month before date of infection, compared to negative controls matched on sex, age and chronic disease. Prescription drug categories are based on the Anatomical Therapeutic Chemical Classification System as defined by the World Health Organization (codes are listed in parenthesis). The analysis was adjusted for body mass index (BMI) (<18.5, 18,5–25,25–30, >30 kg/m^2^, missing) smoking (yes, no, former smoker, missing), alcohol intake (0, 0–7, 7–15, >15 units per week, missing), educational level (none or short, middle, long, missing), patient contact (none, partly, full time, missing), and place of living (Capital Region or Region Zealand). *) Indicates SARS-CoV-2 positive fraction in percent of drug users and no-drug users, respectively. Abbreviations: Adj., adjusted. OR, odds ratio. PCR, polymerase chain reaction. SARS-Cov-2, Severe Acute Respiratory Syndrome Coronavirus-2.
(TIF)

S4 Fig Sensitivity analysis of the odds ratios of SARS-CoV-2 positivity (PCR or ELISA) according to the past six months’ prescription drug use for only participants with submitted questionnaire answers prior to a positive SARS-CoV-2 test. Adjusted odds ratios of having a positive SARS-CoV-2 test according to use of prescription drugs (yes/no) in the six months before date of infection, compared to negative controls matched on sex, age and chronic disease. Only participants with a valid questionnaire answer prior to the date of infection were included. Prescription drug categories are based on the Anatomical Therapeutic Chemical Classification System as defined by the World Health Organization (codes are listed in parenthesis). The analysis was adjusted for body mass index (BMI) (<18.5, 18,5–25,25–30, >30 kg/m^2^, missing) smoking (yes, no, former smoker, missing), alcohol intake (0, 0–7, 7–15, >15 units per week, missing), educational level (none or short, middle, long, missing), patient contact (none, partly, full time, missing), and place of living (Capital Region or Region Zealand). *) Indicates SARS-CoV-2 positive fraction in percent of drug users and no-drug users, respectively. Abbreviations: Adj., adjusted. OR, odds ratio. PCR, polymerase chain reaction. SARS-Cov-2, Severe Acute Respiratory Syndrome Coronavirus-2.
(TIF)

S1 File Adjusted analyses of the odds ratios of severe COVID-19 according to selected prescription drugs and selected potential confounding variables. S1 Table: Antibacterials and chronic disease vs COVID-19 severity. S2 Table: Drugs for obstructive airway disease and chronic disease vs COVID-19 severity. S3 Table: Antibacterials and sex vs COVID-19 severity. S4 Table: Drugs for obstructive airway disease and sex vs COVID-19 severity. S5 Table: Antibacterials and age vs COVID-19 severity. S6 Table: Drugs for obstructive airway disease and age vs COVID-19 severity. S7 Table: Antibacterials and BMI vs COVID-19 severity. S8 Table: Drugs for obstructive airway disease and BMI vs COVID-19 severity. S9 Table: “Any drug” exposure, any chronic disease, age and BMI vs COVID-19 severity.
(DOCX)
