# Supplementary figures and images for: Susceptibility to and severity of SARS-CoV-2 infection according to prescription drug use–an observational study of 46,506 Danish healthcare workers
Source: PLoS One. 2024 Nov 27;19(11):e0311260. doi: 10.1371/journal.pone.0311260 (PMC11602038; doi:10.1371/journal.pone.0311260)

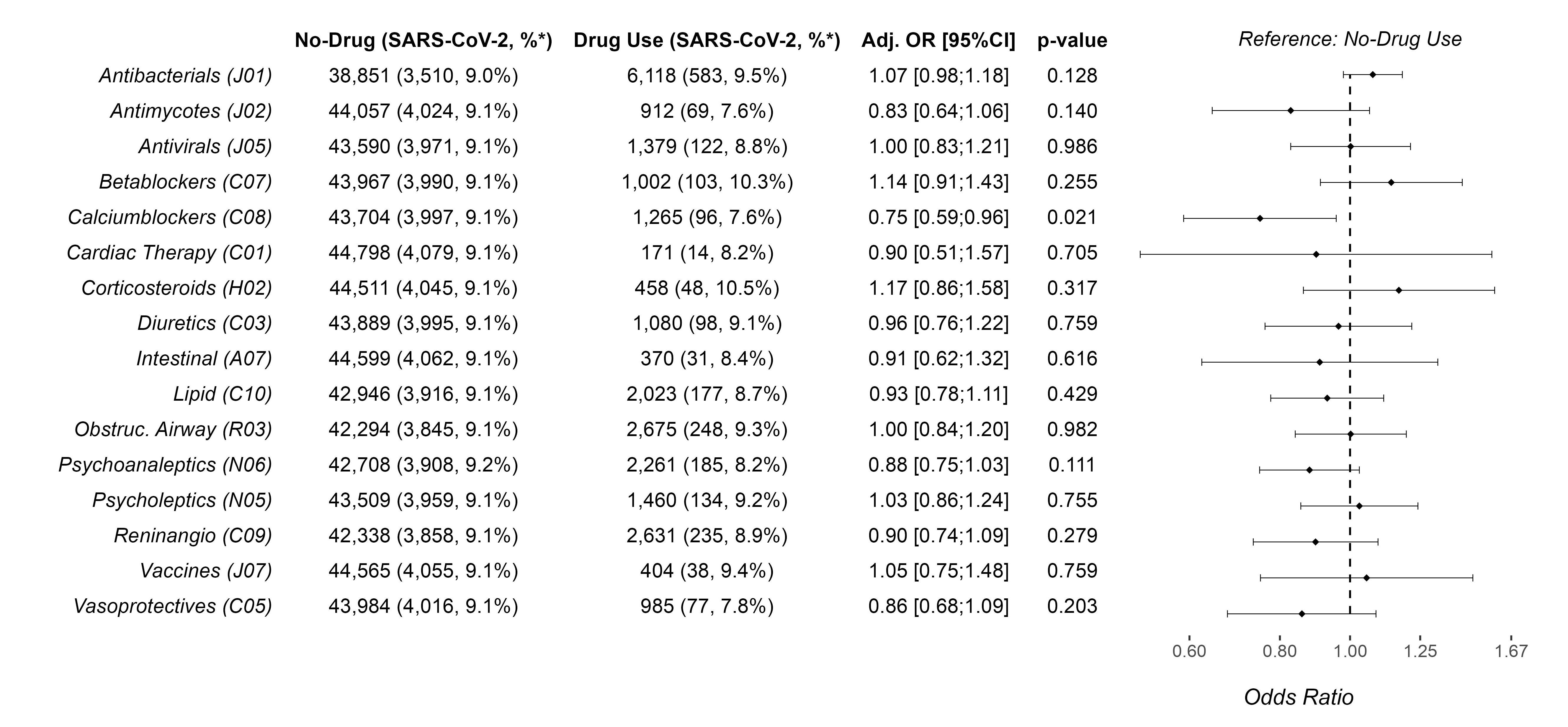

Supplement: S1 Fig — Adjusted odds ratios of having a positive SARS-CoV-2 test (PCR only) according to use of prescription drugs (yes/no) in the six months before date of infection, compared to negative controls matched on sex, age and chronic disease. Prescription drug categories are based on the Anatomical Therapeutic Chemical Classification System as defined by the World Health Organization (codes are listed in parenthesis). The analysis was adjusted for body mass index (BMI) (<18.5, 18,5–25,25–30, >30 kg/m2, missing) smoking (yes, no, former smoker, missing), alcohol intake (0, 0–7, 7–15, >15 units per week, missing), educational level (none or short, middle, long, missing), patient contact (none, partly, full time, missing), and place of living (Capital Region or Region Zealand). *) Indicates SARS-CoV-2 positive fraction in percent of drug users and no-drug users, respectively. Abbreviations: Adj., adjusted. OR, odds ratio. PCR, polymerase chain reaction. SARS-Cov-2, Severe Acute Respiratory Syndrome Coronavirus-2. (TIF) [file pone.0311260.s001.tif]

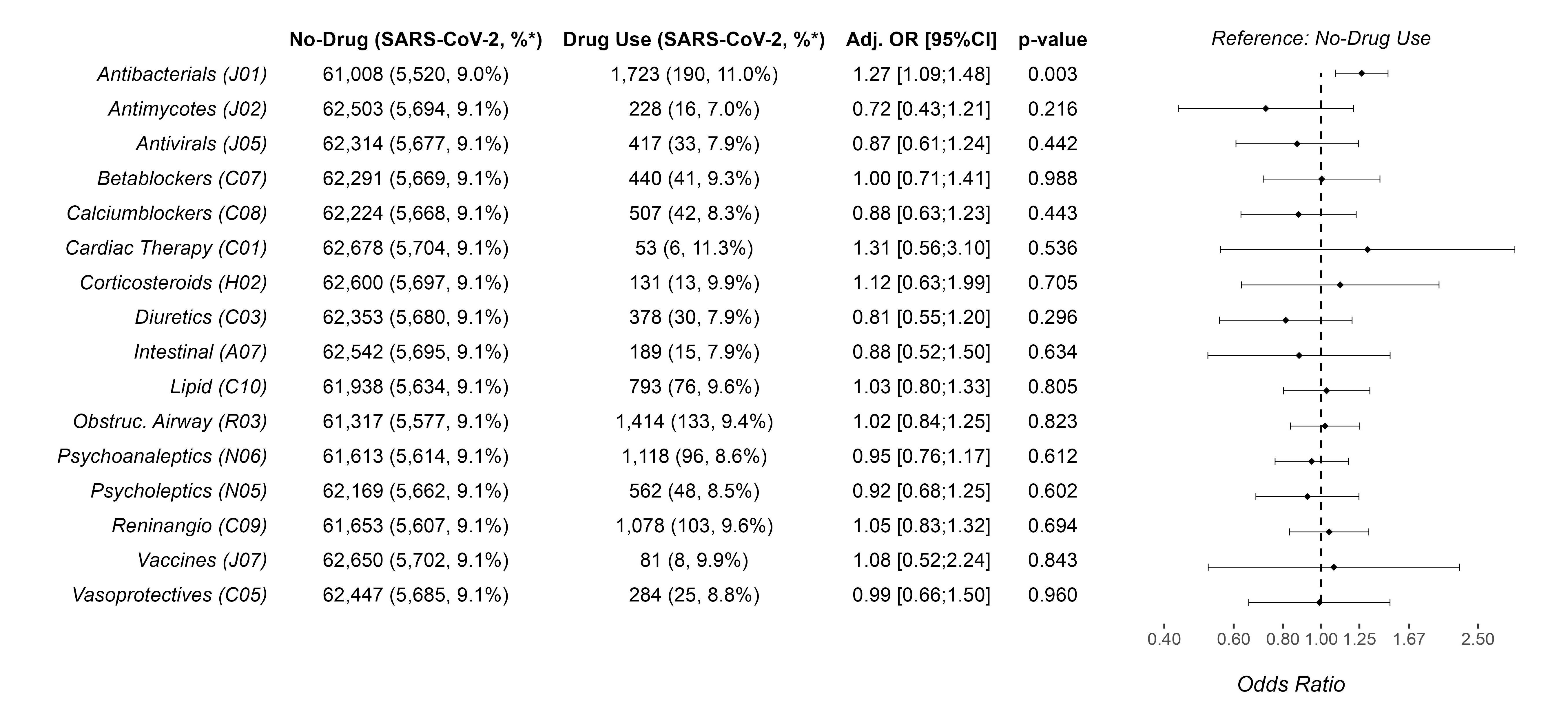

Supplement: S2 Fig — Adjusted odds ratios of having a positive SARS-CoV-2 test (PCR or ELISA) according to use of prescription drugs (yes/no) in the month before date of infection, compared to negative controls matched on sex, age and chronic disease. Prescription drug categories are based on the Anatomical Therapeutic Chemical Classification System as defined by the World Health Organization (codes are listed in parenthesis). The analysis was adjusted for body mass index (BMI) (<18.5, 18,5–25,25–30, >30 kg/m2, missing) smoking (yes, no, former smoker, missing), alcohol intake (0, 0–7, 7–15, >15 units per week, missing), educational level (none or short, middle, long, missing), patient contact (none, partly, full time, missing), and place of living (Capital Region or Region Zealand). *) Indicates SARS-CoV-2 positive fraction in percent of drug users and no-drug users, respectively. Abbreviations: Adj., adjusted. OR, odds ratio. PCR, polymerase chain reaction. SARS-Cov-2, Severe Acute Respiratory Syndrome Coronavirus-2. (TIF) [file pone.0311260.s002.tif]

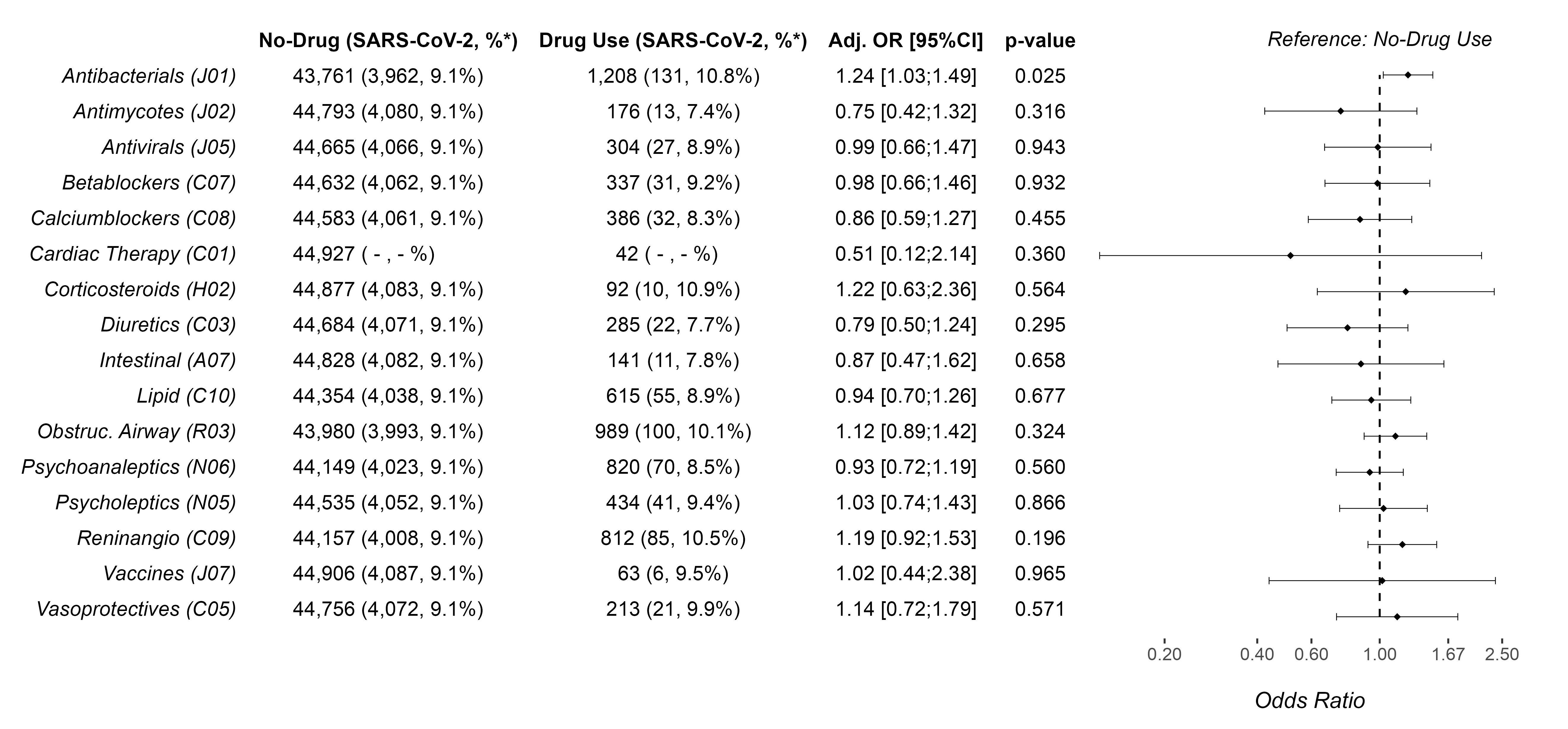

Supplement: S3 Fig — Adjusted odds ratios of having a positive SARS-CoV-2 test (PCR only) according to use of prescription drugs (yes/no) in the month before date of infection, compared to negative controls matched on sex, age and chronic disease. Prescription drug categories are based on the Anatomical Therapeutic Chemical Classification System as defined by the World Health Organization (codes are listed in parenthesis). The analysis was adjusted for body mass index (BMI) (<18.5, 18,5–25,25–30, >30 kg/m2, missing) smoking (yes, no, former smoker, missing), alcohol intake (0, 0–7, 7–15, >15 units per week, missing), educational level (none or short, middle, long, missing), patient contact (none, partly, full time, missing), and place of living (Capital Region or Region Zealand). *) Indicates SARS-CoV-2 positive fraction in percent of drug users and no-drug users, respectively. Abbreviations: Adj., adjusted. OR, odds ratio. PCR, polymerase chain reaction. SARS-Cov-2, Severe Acute Respiratory Syndrome Coronavirus-2. (TIF) [file pone.0311260.s003.tif]

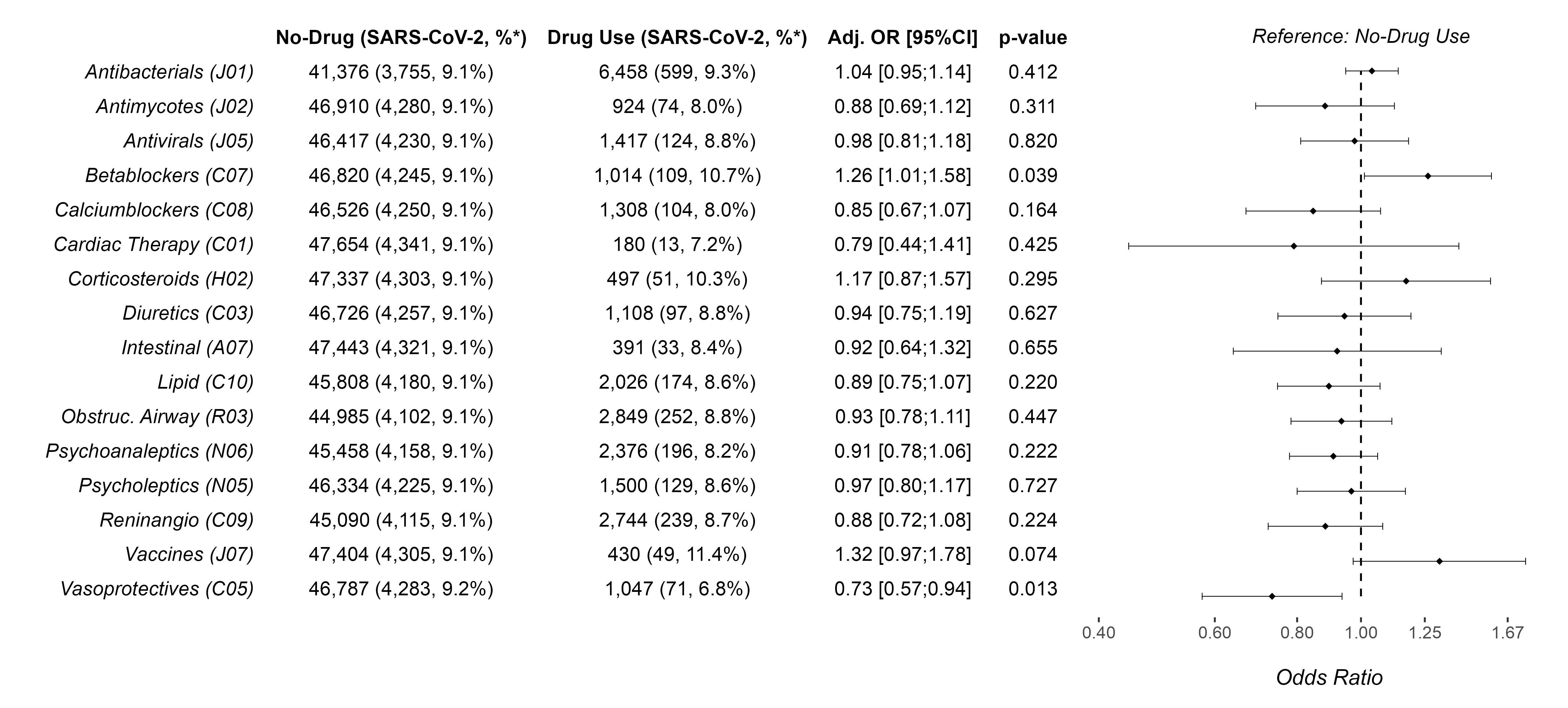

Supplement: S4 Fig — Adjusted odds ratios of having a positive SARS-CoV-2 test according to use of prescription drugs (yes/no) in the six months before date of infection, compared to negative controls matched on sex, age and chronic disease. Only participants with a valid questionnaire answer prior to the date of infection were included. Prescription drug categories are based on the Anatomical Therapeutic Chemical Classification System as defined by the World Health Organization (codes are listed in parenthesis). The analysis was adjusted for body mass index (BMI) (<18.5, 18,5–25,25–30, >30 kg/m2, missing) smoking (yes, no, former smoker, missing), alcohol intake (0, 0–7, 7–15, >15 units per week, missing), educational level (none or short, middle, long, missing), patient contact (none, partly, full time, missing), and place of living (Capital Region or Region Zealand). *) Indicates SARS-CoV-2 positive fraction in percent of drug users and no-drug users, respectively. Abbreviations: Adj., adjusted. OR, odds ratio. PCR, polymerase chain reaction. SARS-Cov-2, Severe Acute Respiratory Syndrome Coronavirus-2. (TIF) [file pone.0311260.s004.tif]
