# Supplementary material for: Susceptibility to and severity of SARS-CoV-2 infection according to prescription drug use–an observational study of 46,506 Danish healthcare workers
Source: PLoS One. 2024 Nov 27;19(11):e0311260. doi: 10.1371/journal.pone.0311260 (PMC11602038; doi:10.1371/journal.pone.0311260)
Supplement: S1 File — S1 Table: Antibacterials and chronic disease vs COVID-19 severity. S2 Table: Drugs for obstructive airway disease and chronic disease vs COVID-19 severity. S3 Table: Antibacterials and sex vs COVID-19 severity. S4 Table: Drugs for obstructive airway disease and sex vs COVID-19 severity. S5 Table: Antibacterials and age vs COVID-19 severity. S6 Table: Drugs for obstructive airway disease and age vs COVID-19 severity. S7 Table: Antibacterials and BMI vs COVID-19 severity. S8 Table: Drugs for obstructive airway disease and BMI vs COVID-19 severity. S9 Table: “Any drug” exposure, any chronic disease, age and BMI vs COVID-19 severity. (DOCX) [file pone.0311260.s005.docx]

# **S1-S9 Tables: Adjusted analyses of the odds ratios of severe COVID-19 according to selected prescription drugs and selected potential confounding variables.**

## Adjustment for chronic disease

**S1 Table: Antibacterials and chronic disease vs COVID-19 severity**

| **Variable** | **Crude OR** | **Crude p-value (95%CI)** | **Adj OR**  **(95%CI)** | **p-value adj** |
| --- | --- | --- | --- | --- |
| J01 antibiotics – exposed vs non-exposed(ref) | 2.74 (1.62, 4.61) | <0.001 | 2.40 (1.48, 4.24) | 0.001 |
| Chronic disease – yes vs no (ref) | 4.02 (2.48, 6.51) | <0.001 | 3.84 (2.37, 6.23) | <0.001 |

**S2 Table: Drugs for obstructive airway disease and chronic disease vs COVID-19 severity**

| **Variable** | **Crude OR** | **Crude p-value (95%CI)** | **Adj OR**  **(95%CI)** | **p-value adj** |
| --- | --- | --- | --- | --- |
| R03 obstructive airway – exposed vs non-exposed(ref) | 4.49 (2.49, 8.08) | <0.001 | 2.31 (1.21,4.42) | 0.011 |
| Chronic disease – yes vs no (ref) | 4.02 (2.48,6.51) | <0.001 | 3.21 (1.89, 5.46) | <0.001 |

## Adjustment for sex

**S3 Table: Antibacterials and sex vs COVID-19 severity**

|  | **crude OR (95%CI)** | **crude P value** | **adj. OR (95%CI)** | **p-value adj** |
| --- | --- | --- | --- | --- |
| J01_antibacterials: exposed vs non-exposed (ref) | 2.74 (1.62,4.61) | < 0.001 | 2.88 (1.71,4.87) | <0.001 |
| Sex – man vs woman(ref) | 2.32 (1.41,3.83) | < 0.001 | 2.45 (1.48,4.04) | 0< 0.001 |

**S4 Table: Drugs for obstructive airway disease and sex vs COVID-19 severity**

|  | **crude OR (95%CI)** | **crude P value** | **adj. OR (95%CI)** | **P(LR-test)** |
| --- | --- | --- | --- | --- |
| R03_obstructiveairway: exposed vs non-exposed (ref) | 4.49 (2.49,8.08) | < 0.001 | 4.50 (2.49,8.12) | < 0.001 |
| Sex – man vs woman(ref) | 2.32 (1.41,3.83) | < 0.001 | 2.33 (1.31,3.84) | 0.002 |

Adjustment for age

**S5 Table: Antibacterials and age vs COVID-19 severity**

|  | **crude OR (95%CI)** | **crude P value** | **adj. OR (95%CI)** | **P(LR-test)** |
| --- | --- | --- | --- | --- |
| J01_antibacterials: exposed vs non exposed | 2.74 (1.62,4.61) | < 0.001 | 2.76 (1.63,4.66) | < 0.001 |
| Age: Below 30 years (ref),  30-50 years, above 50 years | 1.7 (0.63, 4.55)  6.02 (2.38, 15.23) | 0.295  <0.001 | 1.67 (0.62, 4.5)  5.99 (2.37,15.19) | < 0. 001 |

**S6 Table: Drugs for obstructive airway disease and age vs severe COVID-19 severity**

|  | **crude OR (95%CI)** | **crude P value** | **adj. OR (95%CI)** | **P(LR-test)** |
| --- | --- | --- | --- | --- |
| R03_obstructiveairway: exposed vs non exposed | 4.49 (2.49,8.08) | < 0.001 | 4.01 (2.21,7.26) | < 0.001 |
| Age: Below 30 years (ref), 30-50 years, above 50 years | 1.7 (0.63, 4.55)  6.02 (2.38, 15.23) | 0.2.95  <0.001 | 1.61 (0.60,4.33)  6.84 (2.18,14.05) | < 0.001 |

## Adjustment for BMI

**S7 Table: Antibacterials and BMI vs COVID-19 severity**

|  | **crude OR (95%CI)** | **crude P value** | **adj. OR (95%CI)** | **P(LR-test)** |
| --- | --- | --- | --- | --- |
| J01_antibacterials: exposed vs non exposed | 2.74 (1.62, 4.61) | <0.001 | 2.77 (1.64, 4.67) | <0.001 |
| BMI: normal, underweight or missing(ref),  over weight, obese | 1.86 (1.07,3.24)  2.39 (1.29,4.43) | 0.028  0.006 | 1.85 (1.06, 3.23)  2.44 (1.32, 4.54) | 0.01 |

**S8 Table: Drugs for obstructive airway disease and BMI vs COVID-19 severity**

|  | **crude OR (95%CI)** | **crude P value** | **adj. OR (95%CI)** | **P(LR-test)** |
| --- | --- | --- | --- | --- |
| R03_obstructiveairway_exposed vs non exposed | 4.49 (2.49, 8.08) | <0.001 | 4.18 (2.31, 7.55) | <0.001 |
| BMI: normal, underweight or missing(ref),  over weight, obese | 1.86 (1.07, 3.24)  2.39 (1.29, 4.43) | 0.028  0.006 | 1.76 (1.01, 3.07)  2.21 (1.19, 4.12) | 0.026 |

**S9 Table: “Any drug” exposure, any chronic disease, age and BMI vs COVID-19 severity**

|  | **crude OR (95%CI)** | **crude P value** | **adj. OR (95%CI)** | **P(LR-test)** |
| --- | --- | --- | --- | --- |
| Any drug exposure: yes vs no(ref) | 3.48 (2.10,5.78) | < 0.001 | 2.04 (1.16,3.59) | 0.013 |
| Age: Below 30 years (ref), 30-50 years, above 50 years | 1.70 (0.63,4.55)  6.02 (2.38,15.23) | 0.295  < 0.001 | 1.35 (0.50,3.67)  3.63 (1.40,9.46) | < 0. 001 |
| BMI: normal weight (ref), over weight, obese | 1.86 (1.07,3.24)  2.39 (1.29,4.43) | 0.028  0.006 | 1.42 (0.81, 2. 50)  1.56 (0.83, 2.96) | 0.305 |
| Chronic disease: yes vs no (ref) | 4.02 (2.48,6.51) | < 0.001 | 2.16 (1.25,3.75) | 0.006 |
